# Immune–Epigenetic Effects of Environmental Pollutants: Mechanisms, Biomarkers, and Transgenerational Impact

**DOI:** 10.3390/cimb47090703

**Published:** 2025-09-01

**Authors:** Sandeep R Reddy, Manjunatha Bangeppagari, Sang Joon Lee

**Affiliations:** 1 Department of Cell Biology and Molecular Genetics, Sri Devaraj Urs Academy of Higher Education and Research (Deemed to Be University), Tamaka, Kolar 563103, Karnataka, India; 2 Center for Biofluid and Biomimic Research, Pohang University of Science and Technology (POSTECH), Pohang 37673, Republic of Korea

**Keywords:** immune epigenetics, environmental pollutants, DNA methylation, transgenerational inheritance, inflammatory signalling

## Abstract

Environmental pollutants such as heavy metals, endocrine-disrupting chemicals, microplastics, and airborne particulates are increasingly recognized for their potential to influence immune function through epigenetic mechanisms. This review examines conserved pollutant-associated pathways at interfaces of immunity and epigenetics, with particular attention to Toll-like receptor–NF-κB signalling, NLRP3 inflammasome activity, and reactive oxygen species-driven cascades. Evidence from cellular, animal, and epidemiological studies indicates that these pathways may converge on chromatin regulators such as DNA methyltransferases, histone deacetylases, and EZH2, leading to DNA methylation shifts, histone modifications, and altered chromatin accessibility. Pollutants are also reported to modulate non-coding RNAs, including miR-21, miR-155, and several lncRNAs, which can act as intermediaries between cytokine signalling and epigenetic remodelling. Findings from transgenerational models suggest that pollutant-linked immune–epigenetic alterations might persist across generations, raising the possibility of long-term consequences for immune and neurodevelopmental health. Comparative analyses further indicate convergence across diverse pollutant classes, pointing to a shared mechanistic axis of immune–epigenetic disruption. Overall, these insights suggest that pollutant-induced immune–epigenetic signatures may contribute to inflammation, altered immune responses, and heritable disease risks, and their clarification could inform biomarker discovery and future precision approaches in immunotoxicology.

## 1. Introduction

Environmental pollutants pose a pervasive and escalating threat to human health, with increasing evidence linking them to immune system dysregulation and the development of various immune-related disorders. Common contaminants, including heavy metals, organic chemicals, airborne particulates, and endocrine-disrupting compounds, interfere with essential immunological processes, raising susceptibility to infections, promoting chronic inflammation, and predisposing individuals to autoimmune, allergic, and cancerous diseases. Heavy metals such as lead (Pb), cadmium (Cd), mercury (Hg), and arsenic (As) are particularly immunotoxic, impairing both innate and adaptive responses and encouraging autoimmunity and vaccine resistance [1,2]. Similarly, organic pollutants like bisphenol A (BPA), phthalates, and dioxins disrupt cytokine balance, hinder immune cell development, and trigger persistent inflammatory states [2,3].

Air pollution, including diesel exhaust, nitrous oxide, and ozone, has also been strongly linked to immune dysfunction, particularly through the upregulation of pro-inflammatory cytokines and the suppression of humoral responses, thereby increasing the risk of allergic and autoimmune conditions [4,5]. Persistent organic pollutants (POPs) such as polychlorinated biphenyls (PCBs), per- and polyfluoroalkyl substances (PFAS), and dichlorodiphenyltrichloroethane (DDT) exert long-lasting immunotoxic effects and are associated with increased asthma and allergy prevalence in children exposed prenatally [6,7]. Endocrine-disrupting chemicals (EDCs), including 3-methyl-4-nitrophenol (MNP), are known to impair immune tolerance by skewing T helper cell responses, reducing regulatory T cell (Treg) populations, and promoting T helper 17 (Th17) differentiation [8]. Additionally, combustion by-products and volatile hydrocarbons such as benzene, toluene, ethylbenzene, and xylene (BTEX compounds) have been linked to cytotoxicity and immune suppression, particularly among individuals living near industrial facilities [9]. Recent studies have also highlighted pollutants that disrupt gut microbiota, such as pesticides and nanoplastics, as indirect yet potent modulators of immune function, leading to systemic inflammation and increased susceptibility to autoimmune diseases [10].

These diverse pollutants converge mechanistically through the epigenetic–immune axis, a regulatory interface wherein environmental exposures induce heritable, non-genetic alterations in gene expression that modulate immune function. This axis encompasses DNA methylation, histone modifications, and non-coding RNA activity, which together shape immune cell differentiation, cytokine signalling, and memory formation [11,12]. In toxicological contexts, such epigenetic modifications are viewed as “molecular memory” of environmental insults, capable of imprinting long-lasting changes on immune cell identity and reactivity [13,14]. In immunology, these mechanisms are recognized as central to the regulation of T helper cell polarization, Treg stability, and innate immune plasticity [15,16].

From an epidemiological perspective, epigenome-wide association studies (EWAS) have identified pollutant-induced methylation signatures in blood that correlate with immune phenotypes and exposure histories, providing a measurable readout of this axis in human populations [17]. Dysregulation of the epigenetic–immune axis has been linked to a wide spectrum of pathologies, including cancer, allergies, autoimmunity, and chronic infections [18,19]. Notably, many studies support a directional model in which environmental exposures initially trigger immune responses that, in turn, drive epigenetic remodelling. For instance, pollutants such as fine particulate matter that is 2.5 μm or less in diameter (PM_2.5_) and ozone activate pro-inflammatory signalling (e.g., IL-6, TNF-α), leading to ROS generation and subsequent epigenetic modifications at immune gene promoters [20,21]. Maternal exposure to air pollutants during pregnancy has been associated with elevated C-reactive protein (CRP) levels alongside DNA methylation changes in both maternal and fetal immune genes, suggesting that pollutant-induced immune activation may act as an upstream driver of epigenetic remodelling [22].

Moreover, dynamic chromatin remodelling and DNA methylation changes occur during immune activation itself, such as during infection or allergen exposure, highlighting the bidirectional communication between immune signalling and epigenetic machinery [17]. Chronic exposure to redox-active agents, such as dioxins, can even establish “transcriptional memory” through stable histone modifications in inflammatory genes, illustrating how immune responses can imprint long-term epigenetic states [23]. These immune-initiated epigenetic changes are also implicated in pollutant-associated autoimmune diseases, where Th17 bias and Treg depletion are linked to specific methylation alterations [24].

Importantly, these effects are not equally distributed across populations. Pregnant women and children are especially vulnerable to pollutant-induced immune–epigenetic reprogramming due to heightened developmental plasticity and incomplete immune maturation. In utero exposure to pollutants like PM_2.5_, NO_2_, and polycyclic aromatic hydrocarbons (PAHs) alters cytokine profiles, histone marks, and immune cell development with transgenerational consequences [25,26]. DNA methylation changes in key immune genes (e.g., IL4, IFNγ, FOXP3) in cord blood have been correlated with maternal exposure to metals and EDCs [27]. Similarly, children exposed to pollutants such as phthalates or arsenic exhibit altered immune gene methylation and increased risk for asthma, autoimmunity, and allergies [28,29]. Conversely, prenatal exposure to non-pathogenic microbes has been shown to induce protective epigenetic changes, suggesting that early environmental signals can either promote or buffer immune dysregulation [30].

Recent frameworks in exposome research, which define the totality of environmental exposures across the life course, have begun to formally integrate immune and epigenetic components as central mediators of disease risk. Epigenetic changes are now recognized as key mechanisms by which the exposome shapes immune outcomes, particularly in early developmental windows [31,32]. The concept of “trained immunity,” wherein innate immune cells acquire memory-like features via stable epigenetic reprogramming, has been integrated into exposome definitions to explain how early exposures influence later disease susceptibility [33]. In inflammatory bowel disease, for instance, exposome-induced epigenetic modifications of gut mucosal immunity highlight the tripartite interaction between environment, immunity, and chromatin architecture [34,35]. Finally, systems models of the exposome emphasize the interdependence of external exposures, immune signalling pathways, and epigenetic regulators in mediating complex disease phenotypes across the life span [36].

This review consolidates mechanistic evidence on how environmental pollutants, including heavy metals, particulate matter, endocrine disruptors, and microplastics, influence immune function through epigenetic modifications. We also explore the roles of non-coding RNAs, cytokine—chromatin interactions, and transgenerational inheritance in pollutant-induced immune dysregulation. The aim is to establish a mechanistic framework that links immune activation with epigenetic remodelling, providing insights into biomarkers, heritable signatures, and potential avenues for precision immunotoxicology.

## 2. Mechanistic Links from Exposure to Immunotoxicity

Environmental toxicants rarely act as isolated insults; instead, they initiate a cascade of molecular events that begins with immune recognition and leads to durable epigenetic reprogramming. This pollutant–immune–epigenetic axis provides a mechanistic framework for understanding how diverse environmental exposures, ranging from particulate matter and heavy metals to EDCs, induce persistent changes in immune regulation and disease susceptibility. By initiating pro-inflammatory signalling and chromatin remodelling, pollutants not only alter immune phenotypes acutely but also imprint long-term molecular memory into immune cell populations.

At the initiation of this cascade, pollutants are sensed by innate immune receptors, particularly pattern recognition receptors (PRRs) that detect xenobiotics and damage-associated molecular patterns. The aryl hydrocarbon receptor (AHR) is a central node in this network, responsive to a broad class of organic pollutants such as PAHs, dioxins, and halogenated compounds. Upon activation, AHR regulates immune polarization by influencing Th17/Treg balance and suppressing inflammasome activation, linking detoxification to immune homeostasis [37]. Similarly, Toll-like receptor 4 (TLR4) is activated by PM_2.5_, ozone, and other environmental particulates, particularly in alveolar macrophages of the respiratory tract, leading to NF-κB activation and secretion of inflammatory cytokines such as IL-6 and TNF-α [38]. Another sensor, NLRP3, is activated by silica, heavy metals, or reactive oxygen species (ROS) in macrophages and monocytes, forming inflammasomes that drive caspase-1 activation and the release of IL-1β and IL-18, key mediators of chronic inflammation [39]. These sensors serve as the immune system’s first line of defence against pollutants, initiating downstream signalling events with epigenetic consequences.

Once activated, these immune effectors can reprogram the epigenome through direct biochemical signalling. IL-6, for example, downregulates DNMT1 and promotes DNA demethylation, thus sustaining inflammatory gene expression [40]. TNF-α induces histone acetylation at key loci (e.g., H3K9ac, H3K14ac), facilitating chromatin accessibility and myeloid activation [41]. Meanwhile, ROS generated during pollutant metabolism inhibits histone deacetylases (HDACs) and activates TET enzymes, further contributing to hypomethylated, transcriptionally active chromatin states. These immune-derived molecular signals transform transient inflammation into long-lived epigenetic signatures that modulate immune memory, plasticity, and exhaustion.

Not all immune cells respond equally to environmental cues; some exhibit heightened epigenetic plasticity and act as molecular barometers of toxic exposures. Macrophages, particularly during M1 polarization and inflammasome priming, undergo profound chromatin remodelling and can acquire ‘trained immunity’ phenotypes through epigenetic rewiring [42]. Similarly, naive CD4^+^ T cells experience profound epigenetic remodelling during lineage differentiation, where environmental signals (such as cytokines) guide their fate toward Th1, Th2, or Treg lineages. These signals shape lineage-specific epigenetic states by modifying DNA methylation, histone marks, and 3D chromatin architecture to establish distinct gene expression programs [43]. Natural killer (NK) cells, though traditionally considered less plastic, can acquire memory-like properties through cytokine- or virus-driven epigenetic remodelling of chromatin, enabling enhanced recall responses that may extend to toxicant-exposed contexts [44,45,46].

These cellular effects are coordinated by key transcription factors that bridge inflammation and chromatin dynamics. NF-κB, activated downstream of TLRs and cytokine receptors, recruits histone acetyltransferases (HATs) and chromatin modifiers to inflammatory gene loci, reinforcing pro-inflammatory transcriptional loops [47]. Additionally, NF-κB controls the expression of miRNAs such as let-7 and Lin28, which act as post-transcriptional regulators of immune homeostasis. STAT3, often activated by IL-6 and IL-10, promotes chromatin opening and DNA demethylation at genes involved in inflammation and epithelial-to-mesenchymal transition [48]. The JAK/STAT signalling pathways contain intrinsic negative feedback regulators such as SOCS proteins, which limit the persistence of downstream transcriptional responses. As a result, stable epigenetic remodelling is generally established only under conditions of chronic inflammation, when cytokine signalling remains elevated for prolonged periods. Within this context, STAT1 activation via IFNγ has been linked to immune exhaustion and accelerated epigenetic aging in chronic exposure models [49]. These findings highlight how persistent cytokine activity can reshape the epigenome in a lasting manner.

Importantly, several pollutant classes not only trigger inflammatory and epigenetic activity but also exert immunosuppressive effects that impair anti-infective immunity. For example, PAHs suppress both cellular and humoral defences by reducing immunoglobulin levels (IgA, IgG), altering complement activity, and dampening vaccine responses, thereby increasing infection susceptibility [50,51,52]. Heavy metals such as lead, cadmium, and mercury further compromise host defence through epigenetic remodelling of immune genes, impairing lymphocyte differentiation and antiviral responses, and are linked to a greater risk of chronic viral and parasitic infections [53,54]. Similarly, PM_2.5_ and microplastics dysregulate innate and adaptive immune pathways, diminish Treg populations, and disrupt mucosal barriers, collectively reducing resistance to pathogens [8,10,55]. PM_2.5_ modifies cytokine profiles, including IL-1RA and IL-27, and alters histone acetylation marks such as H3K9ac and H3K27ac in immune tissues [26]. Heavy metals like arsenic and cadmium simultaneously induce inflammatory signalling and promote DNA hypomethylation in cytokine gene promoters [56]. EDCs such as BPA and dioxins are known to skew immune polarization toward Th2 and Th17 phenotypes while also modifying epigenetic regulators, including FOXP3 and IL-4 gene loci [57]. Finally, combustion by-products like diesel exhaust particles and PAHs exert dual effects by activating AHR and TLR pathways and remodelling enhancer elements involved in immune gene regulation [58]. Together, these pollutants illustrate the mechanistic interdependence of immune signalling and epigenetic modulation, reinforcing the view that immunotoxicity is inseparable from chromatin remodelling in environmentally exposed populations.

### 2.1. Polycyclic Aromatic Hydrocarbons (PAHs)

PAHs are a group of long-lasting organic pollutants produced by the incomplete combustion of carbon-based substances, frequently present in urban air, cigarette smoke, and industrial discharge. Their immunotoxic effects are mainly exerted through the AHR, a ligand-dependent transcription factor that translates environmental signals into immune responses. When bound to ligands such as benzo[a]pyrene, AHR dimerizes with ARNT (AHR nuclear translocator), moves into the nucleus, and attaches to xenobiotic response elements (XREs), initiating gene expression programs that impact not only detoxification processes but also immune regulation. This includes altered T cell polarization, whereby AHR activation promotes Th17 and Th22 differentiation and impairs Treg function [59,60,61].

PAH exposure has been shown to significantly alter the expression of inflammatory cytokines. In airway epithelial and macrophage models, exposure enhances secretion of IL-1β, IL-18, IL-22, and TNF-α [59,60], while also inducing anti-inflammatory cytokines like IL-10 in certain contexts [61]. In parallel, PAHs modulate microRNA (miRNA) profiles that influence immune gene expression. Notably, exposure to benzo[a]pyrene alters expression of miRNAs targeting IL1B, CXCL14, and CD86 in epithelial and immune cells [62]. Population studies in Bangladesh further show that PAH exposure is associated with altered CD4^+^ T cell subset distribution (Th1, Th2, Th17), mediated in part through miRNA regulation [63]. Supporting transcriptomic evidence from bronchial epithelial cells exposed to PAHs confirms induction of innate immune pathways consistent with epigenetic and miRNA-mediated control [64].

PAHs also disrupt epigenetic regulatory systems, particularly DNA methylation and histone modification. AHR activation and reactive oxygen species (ROS) generated in response to PAHs modulate DNA methyltransferases DNMT1, DNMT3A, and DNMT3B, altering promoter methylation of immune-related genes such as IL-4, IFNγ, and FOXP3 [65]. Concurrently, histone deacetylases (HDAC1, HDAC3, HDAC6) are upregulated, leading to chromatin condensation and repression of immune genes; inhibition of these HDACs restores interferon responses in PAH-exposed models [66,67].

At the chromatin level, PAHs reduce the expression of the AHR repressor (AHRR), leading to sustained AHR activation and transcription of pro-inflammatory genes [68]. Enhancer elements marked by H3K27ac are also remodelled in pulmonary endothelial and epithelial cells following PAH exposure, with transcription factor recruitment (e.g., ERG, FLI1 of the ETS family) that regulates vascular inflammation and remodelling [69,70].

The consequences of this immune–epigenetic remodelling extend to multiple organ systems. In asthma and atopic dermatitis, PAH exposure correlates with Th2-biased cytokine expression and epigenetic modifications at loci such as IL4 and IL13 [71]. Autoimmune pathologies, including rheumatoid arthritis and systemic lupus erythematosus, have been associated with PAH-induced Th17 expansion and silencing of regulatory genes via promoter hypermethylation [72]. In early life, altered DNA methylation patterns induced by PAHs affect T cell lineage decisions, contributing to increased risks for infection and allergic sensitization [73]. Additionally, PAHs promote enhancer remodelling and endothelial activation in vascular tissues, contributing to atherosclerosis and pulmonary hypertension [74]. As established carcinogens, PAHs also silence tumour suppressor genes such as MGMT through DNA hypermethylation and histone modification, promoting oncogenesis [75].

Together, these findings highlight PAHs as prototypical immunotoxicants that induce immune activation and epigenetic dysregulation through well-characterized molecular mediators.

### 2.2. Particulate Matter (PM2.5, PM10)

Particulate matter (PM), particularly fine (PM_2.5_) and coarse (PM_10_) fractions, represents one of the most studied categories of air pollutants concerning immune and epigenetic dysregulation. Upon inhalation or dermal contact, PM engages innate immune receptors such as TLR4 and NOD-like receptor protein 3 (NLRP3), leading to activation of downstream signalling via NF-κB, MAPK, and NRF2 pathways. These cascades collectively mediate the production of inflammatory cytokines, including IL-6, IL-8, IL-1β, and TNF-α, while often suppressing antiviral mediators like interferon-β (IFN-β). In human bronchial epithelial cells, PM_2.5_ exposure has been shown to increase secretion of IL-6 and IL-8 and reduce IFN-β, particularly in contexts of allergen sensitization or co-infection [76]. Diesel exhaust particles (DEPs), a PM subtype, similarly activate MAPK—NRF2 signalling in epithelial and endothelial cells, amplifying IL-8 production and oxidative stress [77]. In the skin, PM exposure induces TLR activation and NLRP3 inflammasome assembly in resident immune cells, resulting in elevated IL-1β levels and perturbed T cell regulation [78].

The inflammatory responses triggered by PM are closely linked to epigenetic remodelling. Oxidative stress induced by PM-derived reactive oxygen species (ROS) acts as a mediator linking immune signalling with changes in the epigenome. Ultrafine particles (PM_0.1_) have been demonstrated to downregulate NRF2 and activate NF-κB, increasing expression of DNA methyltransferases and altering DNA methylation profiles in lung epithelial cells [79]. In macrophages, PM10 has been shown to elicit cytotoxic responses while disrupting DNA methylation in repetitive genomic elements [80]. PM_2.5_ exposure during pregnancy induces histone modifications, including H3K9ac, H3K27ac, and H4K20me1/3, and alters cytokine expression in both maternal and fetal immune tissues, suggesting potential transgenerational impacts [26]. Moreover, ROS-mediated modulation of ten-eleven translocation (TET) enzymes and histone deacetylases (HDACs) results in global hypomethylation and enhanced chromatin accessibility in inflamed tissues [81].

These epigenetic alterations are highly tissue specific. In pulmonary tissues, PM exposure is associated with increased enhancer activity and global hypomethylation, which impairs epithelial barrier function and enhances susceptibility to tumorigenesis. In cardiovascular tissues, PM induces hypomethylation of vascular gene promoters such as NOS3 and ICAM1 and dysregulates HDAC expression and non-coding RNA networks involved in endothelial remodelling [82,83]. This organ-specific epigenetic modulation emphasizes the necessity for context-dependent research on pollutant-induced pathology.

Cumulatively, these immune and epigenetic perturbations contribute to a broad spectrum of chronic diseases. In chronic obstructive pulmonary disease (COPD), PM exposure is associated with altered expression of miRNAs (e.g., miR-146a, miR-21) and hypomethylation of inflammatory gene promoters, which exacerbate disease progression [84]. In asthma, histone acetylation changes and methylation alterations in genes such as IL-4 and FOXP3 promote Th2-dominated immune responses and airway hyperreactivity [85]. Cardiovascular outcomes have also been linked to PM exposure, as evidenced by changes in mitochondrial DNA methylation and elevated levels of circulating cell-free mitochondrial DNA, both indicative of oxidative stress and endothelial dysfunction [86]. In cancer and fibrotic lung disease, PM-induced epigenetic silencing of tumour suppressor genes such as MGMT and P16 has been implicated [87].

These findings position PM as a potent modulator of both immune responses and epigenetic regulation, reinforcing its relevance in the development of immune-mediated and chronic systemic diseases.

### 2.3. Heavy Metals

Heavy metals such as lead (Pb), cadmium (Cd), mercury (Hg), arsenic (As), and nickel (Ni) are prominent environmental toxicants with established immunotoxic and epigenetic effects. These metals modulate immune responses through conserved signalling pathways, notably TLR4–NF-κB activation, redox-sensitive transcriptional circuits, and inflammasome regulation. Lead exposure stimulates the TLR4–NF-κB axis, elevating IL-1β and IL-6 levels while suppressing anti-inflammatory cytokines such as IL-1 receptor antagonist (IL-1RA) [88]. Cadmium triggers both TLR and NLRP3 inflammasome pathways, enhancing secretion of pro-inflammatory cytokines such as TNF-α and IFNγ and driving Th17 polarization [89]. Conversely, mercury and arsenic suppress inflammasome activation and reduce IL-1β expression, indicating immunosuppressive properties in specific contexts [90]. These divergent immunomodulatory profiles reflect metal-specific interactions with innate sensors and cytokine networks.

In parallel, heavy metals like cadmium induce oxidative stress that disrupts histone-modifying enzyme activity, resulting in altered histone acetylation and methylation patterns—including at inflammatory gene promoters such as IL6—thereby facilitating enhanced IL-6 expression in macrophages [91]. Demethylation of IFNγ is linked to increased Th1 activity and chronic immune activation [92,93], while hypermethylation at IFNγ loci in children has been correlated with impaired vaccine responses, suggesting diminished adaptive immunity [94]. These alterations are both exposure specific and gene specific, highlighting the nuanced regulation of cytokine networks by epigenetic marks.

Heavy metals also affect histone post-translational modifications. Lead exposure induces persistent trimethylation at histone H3 lysine 27 (H3K27me3), a repressive mark associated with immune gene silencing [95]. Nickel reduces H3K27me3 and increases H3K4me3, shifting chromatin toward an active configuration that promotes inflammatory gene expression [96]. Arsenic alters histone mark distributions in bronchial epithelial cells, simultaneously repressing tumour suppressor genes and upregulating immune-related oncogenes [97]. These findings suggest that heavy metals can either promote chronic inflammation or facilitate immune evasion, depending on the chromatin context.

Notably, certain epigenetic modifications induced by heavy metals are stably inherited across generations. In zebrafish, mercury exposure resulted in sperm-specific differentially methylated regions (DMRs) that were transmitted to the F2 generation and linked to neuroimmune dysfunction [98]. Similar multigenerational effects have been observed with cadmium, where persistent DNA methylation shifts correspond to adaptive growth and immune traits [99]. In plant systems such as rice, Pb and Cd exposures led to CHG hypomethylation in F3 progeny, associated with increased stress tolerance [100]. These findings raise important considerations regarding the heritability of immune dysfunction from metal exposures, particularly when exposures occur during gametogenesis or fetal development.

The epigenetic potency of heavy metals varies by structure and mechanism of action. Arsenic is among the most potent, capable of inducing both promoter hypermethylation and histone mark redistribution that affect immune genes and non-coding RNA networks [97]. Cadmium disrupts global DNA methylation and DNMT1 activity while also altering histone acetylation [89]. Nickel and lead primarily act through histone methylation and redox mechanisms, whereas mercury’s epigenetic effects are most pronounced during fetal development, often via disrupted DNA demethylation and chromatin remodelling [101]. These mechanistic insights suggest a toxicological hierarchy that should inform risk modelling and regulatory prioritization of heavy metal exposure.

Taken together, heavy metals constitute a mechanistically diverse pollutant class that activates inflammatory pathways and modifies chromatin architecture through both direct and indirect routes. These dual actions converge on immunoregulatory circuits, contributing to chronic disease susceptibility, immune aging, and in some cases, transgenerational immune dysregulation.

### 2.4. Endocrine-Disrupting Chemicals (EDCs)

EDCs such as BPAs, phthalates, dioxins, parabens, and nonylphenols alter immune homeostasis by mimicking or antagonizing endogenous hormones. These compounds interact with nuclear hormone receptors, leading to deviations in cytokine expression and immune cell differentiation. Estrogenic EDCs like BPA and phthalates skew the immune balance toward Th2 polarization by increasing IL-4, IL-5, and IL-13 production, thereby heightening the risk of allergic diseases, including asthma and atopic dermatitis [102]. Additionally, dioxins and BPA activate the aryl hydrocarbon receptor (AHR) and NF-κB pathways, enhancing IL-6 and IL-1β expression, which promotes Th17 responses associated with autoimmunity [103]. Recent evidence indicates that certain EDCs can suppress Th1 immunity, potentially involving IFNγ downregulation and disruption of TLR signalling pathways [104].

Exposure to EDCs has profound implications for immune regulation through epigenetic mechanisms, particularly via miRNA dysregulation. Among the most studied, miR-21 and miR-155 are consistently upregulated in response to EDCs and play central roles in modulating tumour immunosurveillance. These miRNAs drive the expansion of myeloid-derived suppressor cells (MDSCs) by persistently activating the STAT3 pathway as they target its negative regulators. This process fosters an immunosuppressive tumour microenvironment, leading to impaired cytotoxic T cell activity and reduced antitumour responses. In addition, miR-21 downregulates STAT1 signalling in macrophages, inhibiting M1 polarization and promoting tumour-associated macrophage phenotypes that enhance immune tolerance [105,106]. Targeting these miRNAs has been shown to restore antitumour immunity, underscoring their relevance in therapeutic strategies [107].

Beyond miRNAs, EDCs also reshape immune epigenomes through direct interactions with nuclear receptors such as ERα, ERβ, and AHR. Ligand binding by bisphenol analogs (BPA, BPS, BPE) alters DNA methylation and histone modifications at hormone response elements, leading to persistent transcriptional changes. For instance, bisphenol exposure modifies the activity of histone methyltransferases, altering marks such as H3K9me2 and H3K9me3, with evidence of these changes being transmitted across generations [108]. Recent studies also show that bisphenol-induced epimutations can persist through differentiation in pluripotent cells, suggesting partial inheritance of altered chromatin states [109]. While evidence in immune lineages is limited, accumulating data support the hypothesis that EDCs establish a form of epigenetic memory with implications for long-term immune dysfunction and disease susceptibility [110].

EDCs can reprogram immune responses by modulating HDACs, which intersect with NF-κB and STAT3 signalling to enforce immune tolerance. Among HDAC isoforms, HDAC6 has emerged as a pivotal regulator: it forms complexes with STAT3 in antigen-presenting cells (APCs), enabling STAT3 recruitment to the IL-10 promoter and thereby driving IL-10 production, a cytokine central to immunosuppression. Genetic or pharmacological inhibition of HDAC6 disrupts STAT3 phosphorylation and IL-10 synthesis, shifting APCs toward a pro-inflammatory phenotype and restoring T cell responsiveness [111]. Similarly, class I HDACs, particularly HDAC1 and HDAC3, regulate T cell tolerance by restricting chromatin accessibility. Their inhibition enhances NF-κB binding, promotes effector gene expression in CD8^+^ T cells, and fosters memory T cell formation with improved antitumour activity, highlighting them as additional nodes of immune reprogramming [112]. These findings underscore how EDC-mediated HDAC disruption can alter chromatin landscapes, promoting tolerance at the expense of immune surveillance.

Beyond acute reprogramming, EDC-driven epigenetic modifications can persist and, in some cases, be transmitted across generations. DNA methylation changes induced by environmental exposures in germ cells can affect immune progenitors in offspring, modifying susceptibility to infection and autoimmunity [31,113]. While histone modifications and miRNA dysregulation are increasingly recognized as contributors to immune memory and disease risk, direct evidence for their stable inheritance in immune lineages remains limited [11]. Nevertheless, the persistence of EDC-induced epigenetic memory, particularly via DNA methylation, suggests long-term impacts on immunity and raises concerns about multigenerational vulnerability to infection, autoimmunity, and cancer.

Collectively, EDCs exert complex and durable effects on immune signalling and epigenetic programming. Their capacity to modulate immune pathways through hormone receptor activation, miRNA regulation, HDAC inhibition, and histone remodelling underscores their broad impact on immune plasticity and long-term health. Given their persistence in the environment and heightened activity during developmental periods, EDCs remain a high-priority target in immunotoxicology and environmental health research.

### 2.5. Emerging Pollutants

Emerging environmental contaminants, including microplastics (MPs), perfluorooctanoic acid (PFOA), BPA, and phthalates, pose a growing concern due to their immunomodulatory and epigenetic effects. These pollutants activate a range of immune cells, such as macrophages, dendritic cells (DCs), and CD4^+^ T cells through innate immune pathways including the cyclic GMP-AMP synthase (cGAS)-stimulator of interferon genes (STING) axis and NF-κB signalling. In vitro and in vivo studies demonstrate pollutant-induced cytokine shifts, notably increased TNF-α, IL-6, and IFNγ expression, as well as altered macrophage polarization toward regulatory M2-like phenotypes [114,115,116]. In aquatic organisms such as zebrafish, microplastic exposure disrupts mucosal immune functions with altered expression of complement factors and immunoglobulin-related genes.

At the epigenetic level, MPs and associated plasticizers induce both global and locus-specific DNA methylation changes. Exposure to polystyrene MPs results in global hypomethylation in marine invertebrates [117], while mammalian studies highlight alterations in the methylation of immune gene promoters such as IL6 and IFNγ in response to oxidative stress or inflammatory signalling [118,119]. These epigenetic disruptions coincide with impaired DNA repair and altered chromatin accessibility, creating a permissive landscape for prolonged immune activation and genomic instability.

Non-coding RNAs, particularly microRNAs (miRNAs) and long non-coding RNAs (lncRNAs), play a crucial regulatory role in linking immune responses with epigenetic changes triggered by emerging environmental pollutants. Dysregulation of miR-21, miR-155, and miR-146a has been consistently observed in response to nanoplastics and plasticizers, with functional consequences for TLR signalling, cytokine loops, and mitochondrial stress responses [120,121]. Concurrently, lncRNAs such as NEAT1 and HOTAIR modulate inflammatory gene expression through chromatin remodelling and competitive endogenous RNA (ceRNA) activity, amplifying pollutant-induced immunopathology [122].

Critically, there is growing evidence that the immune–epigenetic effects of these pollutants may be heritable. Multigenerational studies in fathead minnows demonstrated that parental exposure to polyethylene MPs resulted in DMRs in F1 progeny, particularly at genes involved in estrogen and immune pathways [123]. Rodent models have shown that BPA and phthalates induce persistent germline methylation changes associated with immune and reproductive dysregulation [124]. Human cohort models conceptually support this heritability, particularly when exposure occurs during hormonally sensitive developmental windows [125].

For a comprehensive evaluation of emerging pollutant effects, an integrated strategy combining in vitro and in vivo models is essential. Primary human immune cells and immune-competent organoid systems offer mechanistic insights into miRNA expression, histone marks, and cytokine outputs [126,127]. Complementary in vivo studies in zebrafish, rodents, and fathead minnows enable assessment of systemic, developmental, and transgenerational immune impacts [123]. Regulatory bodies increasingly utilize these multilayered testing strategies to inform risk assessments and toxicological thresholds [128].

Overall, emerging pollutants modulate immune function and reprogram the epigenome through complex and often heritable mechanisms. These pollutants engage innate immune sensors, alter cytokine and miRNA profiles, and reshape chromatin landscapes in both somatic and germline compartments. Their persistent and tissue-specific effects underscore the importance of integrating immune–epigenetic endpoints into environmental risk assessment frameworks.

## 3. Transgenerational Epigenetic Inheritance of Immune Alterations

Pollutant exposure can induce immune–epigenetic modifications in individuals and further pass these molecular changes to succeeding generations through germline transmission. This process, termed transgenerational epigenetic inheritance (TEI), is increasingly recognized in immunotoxicology, especially in scenarios where maternal immune activation (MIA) during pregnancy alters the fetal epigenetic landscape.

Cytokines such as IL-6, IL-1β, and TNF-α released during MIA can cross the placenta and induce epigenetic modifications in developing fetal tissues, especially in the immune and neural compartments via DNA methylation, histone marks like H3K27me3, and non-coding RNA modulation [129]. These changes lay the foundation for neuroimmune disorders, including schizophrenia, autism spectrum disorder, and depression.

Experimental studies have robustly supported these mechanisms. In a murine MIA model, ref. [130] found that inflammatory exposure during pregnancy induced Nurr1 promoter hypermethylation in the sperm of F1 males, a modification retained into F2 and F3 generations. Complementary behavioural studies by [131] showed that MIA-exposed F1 females exhibited impaired maternal care, leading to altered hippocampal gene expression in F2 offspring, illustrating both molecular and behavioural inheritance.

Evidence from aquatic and mammalian models confirms TEI following pollutant exposure. For example, fathead minnows exposed to polyethylene microplastics (PE-MPs) showed DMRs in F1 offspring at estrogen and immune-regulatory loci [123]. Cadmium exposure has also been reported to induce methylation changes in the cep19 gene that were sustained across four zebrafish generations [99]. Similarly, airborne particulate matter exposure has been associated with heritable changes in germline mitochondrial DNA methylation and oxidative stress responses [132].

Mechanistically, pro-inflammatory cytokines such as IFNγ, IL-1β, and TNF-α help establish epigenetic memory that resists embryonic reprogramming by modulating enzymes like DNMT3A, TET1, and histone acetyltransferases [133,134]. These cytokine-mediated programs contribute to trained immunity and long-term alterations in immune cell fate through sustained chromatin changes.

The sperm epigenome also plays a central role in transmitting pollutant-induced signals. Pathogen exposures such as Toxoplasma gondii infection modify sperm DNA methylation, histone retention, and non-coding RNAs (e.g., miR-34, tsRNAs) that can recapitulate immune and behavioural phenotypes in naïve zygotes [135,136]. Though data on placental miRNAs remain limited, their dysregulation in response to maternal inflammation underscores their likely role in fetal immune programming.

Supportive observational data in humans further link prenatal pollution exposure to persistent epigenetic immune changes. For example, refs. [137,138] demonstrated that air pollution exposure during pregnancy led to lasting DNA methylation shifts at immune loci like IL6 and AHRR in newborns, correlating with increased asthma risk in childhood. Table 1 below summarizes the key experimental models and mechanisms demonstrating TEI across species and pollutant types.

## 4. Cross-Pollutant Integration and Comparison

Despite wide-ranging structural diversity, major environmental pollutant classes including MPs, heavy metals, particulate matter, and plasticizers consistently converge on a core set of immune–epigenetic signalling pathways. These conserved molecular signatures involve the activation of inflammatory pathways (e.g., TLR4, NF-κB, MAPK, and NLRP3) and oxidative stress axes (e.g., ROS–NRF2), which interface with key epigenetic enzymes such as DNMTs, EZH2, HDAC2, and PRMT5. The convergence of these pathways explains how chemically distinct exposures elicit comparable immunotoxic and epigenetic outcomes and helps unify observations from pollutant-specific studies into a broader mechanistic framework.

A prime example of shared immune activation is the TLR4–NF-κB axis, which is triggered by metals, PM, and MPs. These pollutants function as damage-associated molecular patterns (DAMPs) or promote their release, stimulating pro-inflammatory cytokines such as IL-6, IL-1β, and TNF-α [139]. Similarly, pollutants like BPA and PM2.5 amplify these effects by activating NF-κB and MAPKs, which modify chromatin at cytokine gene promoters through histone acetylation [140].

Parallel activation of the NLRP3 inflammasome is another shared feature, especially evident in exposures to PM_2.5_, Cd, Pb, and MPs. This results in caspase-1**–**dependent IL-1β maturation and links to promoter demethylation at the IL1B locus, notably in activated macrophages [141]. Additionally, nearly all pollutant classes elevate ROS, which directly cause oxidative DNA damage and epigenetic instability. In response, NRF2 acts as a key feedback regulator, inducing antioxidant defences while also mediating pollutant-driven epigenetic changes such as altered DNA methylation and histone modifications [139].

These inflammatory cascades converge on common epigenetic effectors. For instance, EZH2, a histone methyltransferase in the PRC2 complex, is upregulated by organochlorines and plasticizers, enhancing H3K27me3 deposition and silencing immune-regulatory genes such as miR-148a [142]. EZH2 can also induce DNMT3A, linking histone repression to de novo DNA methylation at chemokine loci like CCL22/CCR4 [143]. Furthermore, HDAC2, a chromatin repressor, works synergistically with EZH2 to silence immune genes by deacetylating histones and promoting metabolic mediators like PDK1 [144]. PRMT5, another conserved effector, methylates H4R3me2s and regulates immune transcription, particularly in response to endosulfan and related toxins [142].

These cross-pollutant effects are now being modelled using systems immunotoxicology frameworks, where pollutant type, cytokine profile, and epigenetic target are mapped into mechanistic trajectories. For example, PM2.5 and ozone (O_3_) increase IL-33 and Th17 cytokines, which regulate TET1 and demethylate immune gene promoters [81]. BPA and DEHP, by inducing NF-κB and ROS, enhance EZH2 and histone acetyltransferases [145]. Similarly, NO_2_ and SO_2_ elevate IL-6 and IL-1β, upregulating DNMT3A and promoting CpG methylation at immune loci [146].

Common biomarkers across pollutant classes include CpG methylation changes at immune loci such as IL6, IFNγ, and AHRR, as well as non-coding RNAs like miR-21 and miR-155 [147,148]. Enzymes such as DNMT1, EZH2, and TET1 are central mediators of pollutant-induced epigenetic reprogramming, supporting their potential as mechanistic biomarkers in environmental health research [149,150].

However, the field faces notable methodological limitations. Most studies treat exposure, immune phenotype, and epigenetic state in isolation. Although novel methods such as DAR-ChIPEA [151] and AOP-based frameworks [152] attempt to map these relationships, their cross-class applications remain limited. Similarly, exposome studies often lack cell-type resolution and rely on bulk-level data, underscoring the need for single-cell methylome, ChIP-seq, and lncRNA-integrated assays [153]. To illustrate these convergences, Table 2 presents a comparative map of molecular pathways and epigenetic targets commonly engaged by distinct pollutant classes.

## 5. Future Research and Clinical Outlook

Validation of epigenetic biomarkers that reliably reflect pollutant burden and immune disruption should be prioritized in future studies. MicroRNAs such as miR-21, miR-155, and miR-146a are consistently altered by EDCs, tobacco smoke, and air pollution exposures, and their levels often correlate inversely with methylation at immune loci, including IL6, IFNγ, and AHRR [147,154,155]. Genes like GRB2 and PRKCA exhibit coordinated miRNA downregulation and CpG hypomethylation following pollutant exposure, suggesting these loci as key nodes of immune–epigenetic deregulation [154].

Large-scale exposome studies such as HELIX have shown that prenatal cadmium and tobacco exposure induce persistent DNA methylation changes, while childhood exposures affect transcriptomic and metabolomic layers [156]. Computational frameworks like DAR-ChIPEA now enable causal inference by integrating chromatin accessibility with transcription factor activity; such tools have identified C/EBPβ and Rela as mediators of PM_2.5_-induced inflammation [151]. IL-33 and Th17 cytokines have been mechanistically linked to TET1-mediated promoter demethylation in asthma models [81].

Clinically, pollutant-induced epigenetic changes in early life are associated with elevated risk for asthma, neurodevelopmental disorders, and metabolic syndrome [157]. CpG methylation markers like AHRR and IL-6 are already proposed for use in exposure surveillance [158], and immune–epigenetic sensitivity in children has prompted calls for pediatric-specific exposure limits [159]. Importantly, some of these changes are reversible: B vitamin and flavonoid supplementation can restore methylation and reduce inflammatory gene expression [160] and behavioural interventions can partially reverse stress-induced epigenetic marks [161].

Therapeutic strategies targeting this axis include IL-33 inhibitors, HDAC and DNMT inhibitors, and nutritional interventions that modulate TET enzymes and histone modifiers [81,162]. Integrating individual exposome profiles with molecular immune data offers a foundation for precision environmental medicine, mainly in vulnerable populations such as children, pregnant women, and those in high-pollution environments [163].

## 6. Conclusions

Common environmental pollutants such as endocrine-disrupting compounds, heavy metals, MPs, and particulate matter frequently stimulate immune pathways like TLR4–NF-κB, inflammasome activation (NLRP3), and ROS-mediated signalling. These immune activations subsequently induce epigenetic alterations in immune cells, altering DNA methylation patterns, histone modifications, and regulation by non-coding RNAs.

This immune–epigenetic remodelling contributes to chronic inflammatory diseases, immune suppression, autoimmunity, and cancer. In several cases, these changes persist beyond the exposure window and are heritable across generations.

Key biomarkers such as IL6 and IFNγ methylation, miR-21, miR-155, and histone marks like H3K27me3 consistently reflect pollutant-induced immune reprogramming. Despite these advances, mechanistic studies with single-cell resolution and causal frameworks remain limited.

Future work should focus on integrative multi-omics, validated biomarkers, and targeted interventions to mitigate long-term immune–epigenetic consequences of pollutant exposure.

## Figures and Tables

**Table 1:** Experimental Models Demonstrating Transgenerational Epigenetic Inheritance of Immune–Epigenetic Traits.

Model/System	Pollutant or Trigger	Epigenetic Mechanism	Generations Affected	Immune/Neuro Outcome	Reference
Mouse (MIA model)	Maternal immune activation	↑ H3K27me3, sperm *Nurr1* promoter hypermethylation	F1–F3	Neuroimmune dysregulation	[130]
Mouse (behavioural)	MIA → altered maternal care	Hippocampal gene expression dysregulation	F1–F2	Depressive-like behaviour	[131]
Zebrafish	Cadmium	Sustained DNA methylation at *cep19*	F1–F4	Immune-metabolic alterations	[99]
Fathead minnow	Polyethylene microplastics (PE-MPs)	Germline DMRs in immune and hormone response genes	F1	Epigenetic immune reprogramming	[123]
Human (theoretical)	EDCs, metals, air pollution	DNA methylation, histone retention, ncRNA changes	F2–F3 (projected)	Allergy, immune dysregulation	[125]
Cross-species	Airborne particulate matter (PM)	Germline oxidative stress, mtDNA methylation	Transgenerational	Chronic immune activation	[132]
Mouse/epidemiologic	IFNγ, IL-1β, TNF signalling	CpG methylation, histone acetylation	F1–F3	Epigenetic memory of cytokine responses	[133,134]
Mouse	Paternal infection (*T. gondii*)	Sperm DNA methylation, histone retention	F1–F2	Immune activation, behavioural changes	[135]

Note: “→” indicates “leads to” or “results in”; “↑” indicates "increase".

**Table 2:** Comparative Molecular Pathways and Epigenetic Targets Across Pollutant Classes.

Molecular Axis	Pollutants Involved	Key Effectors	Epigenetic Impact	References
TLR4–NF-κB signalling	MPs, metals, air pollution	IL-6, TNF-α, IL-1β	DNMT3A activation; ↑ H3 acetylation	[139,140]
NLRP3 inflammasome	PM2.5, MPs, Cd, Pb	IL-1β, Caspase-1	↓ IL1B promoter methylation	[141]
ROS–NRF2 axis	All pollutant classes	NRF2, IL-6, IL-1β	Oxidative DNA damage; global hypomethylation	[139]
EZH2–DNMT3A–HDAC2 pathway	Endosulfan, BPA, DEHP, organochlorines	miR-148a, CCL22, PDK1	↑ H3K27me3; ↑ CpG methylation; ↓ histone acetylation	[142,143,144]
TET1-mediated demethylation	PM2.5, O_3_, IL-33	Th17 cytokines	↓ Methylation at immune regulatory genes	[81]
miRNA dysregulation	Plasticizers, MPs, air pollutants	miR-155, miR-21, lncRNAs	Cytokine feedback; chromatin remodelling	[147,148]

Note: “↑” indicates “increase”; “↓” indicates “decrease”.

## Data Availability

Not applicable.

## Abbreviations

AHR	Aryl hydrocarbon receptor
AOP	Adverse outcome pathway
ARNT	AHR nuclear translocator
Acetyl CoA	Acetyl coenzyme A
BPA	Bisphenol A
BPS	Bisphenol S
DEHP	Di(2-ethylhexyl) phthalate
DEP	Diesel exhaust particle
DMR	Differentially methylated region
DNMT	DNA methyltransferase
EDC	Endocrine-disrupting chemical
EWAS	Epigenome-wide association study
HAT	Histone acetyltransferase
HDAC	Histone deacetylase
IFN-β	Interferon beta
IFNγ	Interferon gamma
IL	Interleukin
KEAP1	Kelch-like ECH-associated protein 1
MAPK	Mitogen-activated protein kinase
MHC	Major histocompatibility complex
MIA	Maternal immune activation
NF-κB	Nuclear factor kappa-light-chain-enhancer of activated B cells
NLRP3	NOD-like receptor family pyrin domain containing 3
NRF2	Nuclear factor erythroid 2–related factor 2
PAH	Polycyclic aromatic hydrocarbon
PBMC	Peripheral blood mononuclear cell
PE-MP	Polyethylene microplastic
PM	Particulate matter
PRR	Pattern recognition receptor
ROS	Reactive oxygen species
SAH	S-adenosyl homocysteine
SAM	S-adenosyl methionine
STAT	Signal transducer and activator of transcription
TET	Ten-eleven translocation enzyme
TLR	Toll-like receptor
TLR4	Toll-like receptor 4
TNF-α	Tumour necrosis factor alpha
Th	T helper cell
Treg	Regulatory T cell
lncRNA	Long non-coding RNA
miRNA	MicroRNA
